# First person – Sijie Tan and Wen Han Tong

**DOI:** 10.1242/dmm.050130

**Published:** 2023-03-10

## Abstract

First Person is a series of interviews with the first authors of a selection of papers published in Disease Models & Mechanisms, helping researchers promote themselves alongside their papers. Sijie Tan and Wen Han Tong are co-first authors on ‘ Impaired episodic-like memory in a mouse model of Alzheimer's disease is associated with hyperactivity in prefrontal–hippocampal regions’, published in DMM. Sijie conducted the research described in this article while a postdoc in Ajai Vyas's lab at Nanyang Technological University, Singapore. She is now a postdoc in the lab of Nora Kory at Harvard University, Boston, MA, USA, investigating the pathobiology of age-related brain disorders. Wen Han Tong is a postdoc in the lab of Ajai Vyas at Nanyang Technological University, Singapore, investigating neurobiology and translational neuroscience to find interventions for brain diseases.


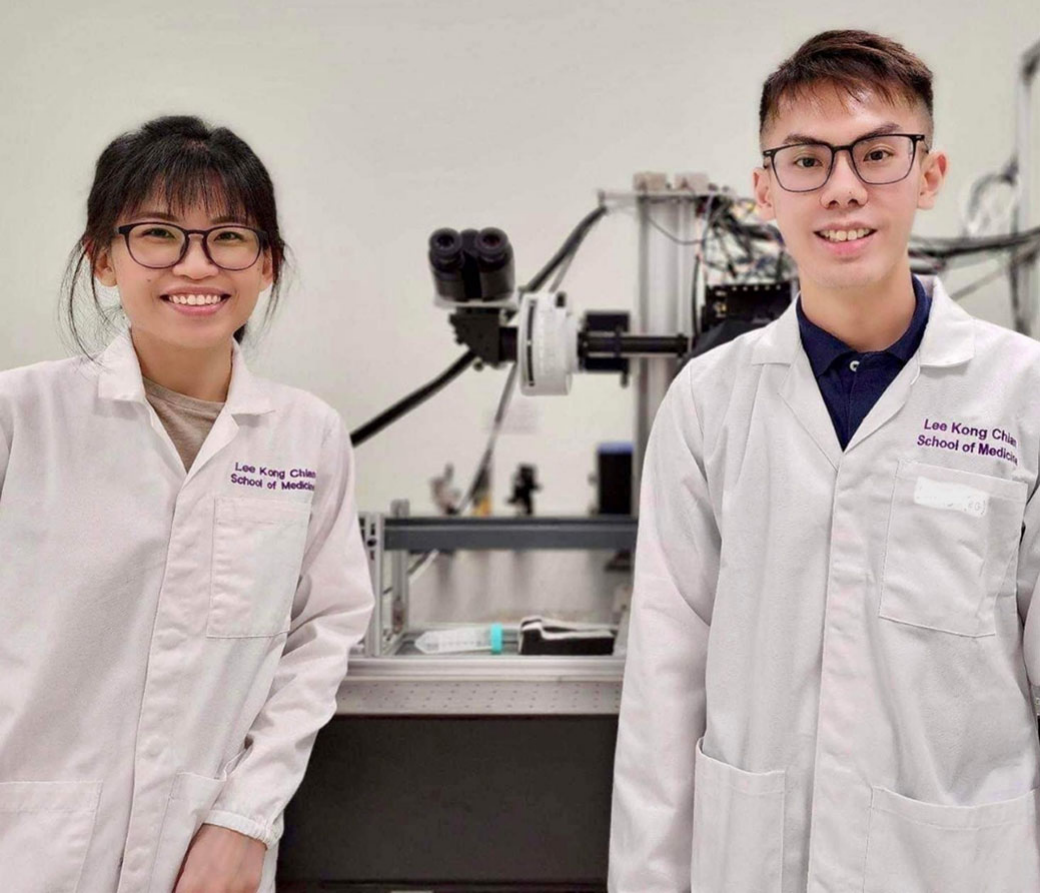


**Sijie Tan (left) and Wen Han Tong (right)**

**How would you explain the main findings of your paper to non-scientific family and friends?**

**ST and WHT:** Imagine having difficulty remembering what you did last Friday. One may dismiss it as a common memory lapse. However, emerging evidence now suggests that a decline in episodic memory, or the inability to remember past events, is an early sign of Alzheimer's disease (AD). Using a mouse model (known as the APP^NL-G-F^ knock-in mouse model) to study the preclinical stage of AD – the so-called early stage when preliminary signs may appear without significant symptoms – our work confirms that episodic memory loss is an early sign of AD. The early episodic memory impairment is also associated with increased brain activity, particularly in regions important for episodic memory. These findings will give researchers and clinicians an idea of ways to identify individuals at risk of developing AD at the early disease stage – the inability to remember past episodes and hyperactivity in specific brain regions are potential diagnostic cues. The ability to identify patients during the preclinical phase means that timely interventions can take place to stop disease progression. Treatments are most effective when administered during preclinical AD before the brain cells start to die and dementia sets in. One may argue that memory losses are normal in life. Especially as we age, forgetfulness becomes a common problem. While our work shows that this is true – episodic memory declines in healthy mice as they age – the memory loss becomes further aggravated in older mice prone to developing AD.

“[…] the use of memory tasks that require greater cognitive load (such as episodic memory) could more accurately characterize individuals at risk of developing AD.”

**What are the potential implications of these results for your field of research?**

**ST and WHT:** First, the complexity of the behavioural task can determine its sensitivity to capture cognitive changes. The lack of discernible memory changes, particularly in the preclinical stage, sometimes does not reflect the lack of deficits. Instead, experimental paradigms may not be sensitive enough to detect the changes. Our work suggests that the use of memory tasks that require greater cognitive load (such as episodic memory) could more accurately characterize individuals at risk of developing AD. Next, our work supports episodic memory decline as an early cognitive hallmark of AD and neuronal hyperactivity as a putative mechanism driving the deficit. These findings suggest that measurement of episodic memory, or clinical imaging for aberrant neuronal activity in the prefrontal–hippocampal regions, could be prognostic tools for AD.

**What are the main advantages and drawbacks of the experimental system you have used as it relates to the disease you are investigating?**

**ST and WHT:** The APP^NL-G-F^ knock-in mouse model recapitulates multiple features of human AD but these mice do not suffer from neurodegeneration. Hence, the APP^NL-G-F^ knock-in mouse model is an excellent tool to study the incipient pathologies of preclinical AD and to identify early disease biomarkers. However, the absence of tau pathology and neurodegeneration in the APP^NL-G-F^ knock-in mice means that they are not suitable to study the later stages of AD or to monitor the biological or cognitive trajectory of the entire AD developmental spectrum.

**Figure:**
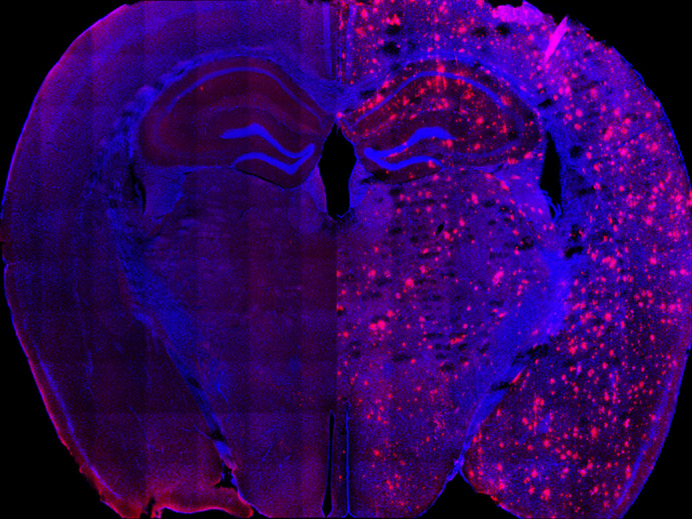
**Amyloid-β is a hallmark of Alzheimer’s disease.** APP^NL-G-F^ mice (right) accumulate massive amounts of amyloid-β (red) in the brain compared to control mice (left).

**What has surprised you the most while conducting your research?**

**ST and WHT:** The fact that we can detect a deficit in episodic memory at an early stage in young APP^NL-G-F^ knock-in mice. Before this, we investigated memory deficits in these mice using classical novelty-based tasks. However, we did not observe any discernible impairments. Subsequently, we switched to a more complex episodic memory task and successfully detected a deficit in memory recall in young APP^NL-G-F^ mice, a phenotype that was otherwise masked. The findings further reinforce the idea that using a task that places a higher demand on cognition can sensitively reveal subtle memory alterations during the presymptomatic AD phase.

**What do you think is the most significant challenge impacting your research at this time and how will this be addressed over the next 10 years?**

**ST and WHT:** To diagnose AD early before the onset of serious symptoms and the availability of diagnostic tools to accurately detect the disease early at a low cost. To address these challenges, computational science and artificial intelligence can be applied to clinical data to identify biomarkers – blood and fluid biomarkers are especially ideal as they are easy to sample. Population-based cohort studies can evaluate the diagnostic potential of a biomarker. In fact, blood plasma levels of amyloid-β, tau and inflammatory cytokines are emerging as biomarkers, and we are expecting more follow-up research on these strategies.

**What changes do you think could improve the professional lives of scientists?**

**ST:** As someone who is on the ‘postdoc treadmill’, one thing I am struggling with is career prospects. I have seen peers getting stuck doing multiple rounds of postdocs. This is a real problem plaguing postdocs committed to research but not yet ready to become faculty. After a while, postdocs get tired out and become unmotivated with the seeming absence of progression in sight. There is a need for more ‘mid-career’ opportunities for postdocs who love research but are not ready to head the lab. The availability of small funding for postdocs to take on ‘mini’ independent roles to run a project, while still under the guidance of a mentor investigator, can help to develop a research career. More emphasis on staff-scientist roles – i.e. allowing postdocs to explore managerial or administrative aspects of research – can further broaden their horizon. Fellowships for postdocs to go overseas to learn new skills is another way to motivate postdocs in their career.

**WHT:** In academic research, the pressure to publish in top-tier journals is immense and has been used as a yardstick to determine the success of a scientist. In some institutions, you are rejected from a junior position or postgraduate scholarship because you do not have top-tier journals in your CV. I believe more opportunities should be given to junior scientists for them to reach their full potential; this comes with having realistic expectations of junior scientists and more emphasis on their ability to conduct quality research. In this highly intense and result-driven environment, it is imperative to consider and develop the mental and emotional wellness of scientists. If not, we will continue to see a trend for brain drain in academia.

**What's next for you?**

**ST:** I have been awarded an A*STAR International Fellowship and just started my 2-year postdoc training at the Harvard T.H. Chan School of Public Health. My research will dive deeper into the root causes of common age-related brain disorders to investigate how changes to mitochondrial physiology contribute to disease development. People have asked what's next after my postdoc at Harvard. Well, I am staying in science but keeping my options open. You never know what opportunities could come your way!

**WHT:** Our findings are exciting and may potentially provide an avenue to rescue memory recall deficit. It will be interesting to investigate if optogenetic silencing of medial prefrontal cortex neurons in APP^NL-G-F^ knock-in mice during the recall task restores episodic-like memory retrieval. This will be the next step I will take, continuing to develop this area of dementia research and building on our findings. Hopefully, something ground-breaking may surface soon.
